# The paleobiology and paleoecology of South African *Lystrosaurus*

**DOI:** 10.7717/peerj.10408

**Published:** 2020-11-24

**Authors:** Jennifer Botha

**Affiliations:** 1 Department of Karoo Palaeontology, National Museum, Bloemfontein, Free State, South Africa; 2 Department of Zoology and Entomology, University of the Free State, Bloemfontein, Free State, South Africa

**Keywords:** Bone histology, Bone microanatomy, Therapsida, Dicynodontia, End-permian mass extinction

## Abstract

*Lystrosaurus* was one of the few tetrapods to survive the end-Permian mass extinction (EPME), the most catastrophic biotic crisis in Phanerozoic history. The significant increased abundance of this genus during the post-extinction Early Triassic recovery period has made *Lystrosaurus* an iconic survivor taxon globally and ideal for studying changes in growth dynamics during a mass extinction. There is potential evidence of a Lilliput effect in *Lystrosaurus* in South Africa as the two Triassic species that became highly abundant after the EPME are relatively smaller than the two Permian species. In order to test this hypothesis a detailed examination of the body size and life history of Permo-Triassic *Lystrosaurus* is required. In this study, the basal skull length and growth patterns of the four South African *Lystrosaurus* species from the Karoo Basin, *L. maccaigi*, *L. curvatus*, *L. murrayi* and *L. declivis*, were examined using cranial measurements and bone histology. The basal skull length measurements show that the Triassic species are smaller than the Permian species and supports previous studies. The osteohistology examination of all four species reveal rapidly forming fibrolamellar bone tissues during early to mid-ontogeny. Growth marks are common in *L. maccaigi* and *L. curvatus*, but rare and inconsistent in the purely Triassic *L. murrayi* and *L. declivis*. The inconsistency of the growth marks in these latter two taxa suggests the presence of developmental plasticity. This feature may have been advantageous in allowing these species to alter their growth patterns in response to environmental cues in the post-extinction Early Triassic climate. An overall transition to slower forming parallel-fibered bone is observed in the largest individuals of *L. maccaigi*, but absent from the limb bones of the other species. The absence of such bone tissue or outer circumferential lamellae in *L. curvatus*, *L. murrayi* and *L. declivis* indicates that even the largest collected specimens do not represent fully grown individuals. Although *L. murrayi* and *L. declivis* are smaller in size, the lack of a growth asymptote in the largest specimens indicates that adult individuals would have been notably larger and may have been similar in size to large *L. maccaigi* and *L. curvatus* when fully grown. Thus, the previously described Lilliput effect, recognized by some authors in the Karoo fossil record (such as the therocephalian *Moschorhinus kitchingi*), may be a product of high juvenile excess mortality in the Triassic rather than a strict “dwarfing” of *Lystrosaurus* species. The lifestyle of *Lystrosaurus* was also re-examined. Although previous studies have proposed an aquatic lifestyle for the genus, the similar morphology and bone microanatomy to several other large terrestrial Permo-Triassic dicynodonts supports a fully terrestrial mode of life.

## Introduction

The “Lilliput effect” was first defined by Urbanek (1993) to describe reduced body sizes in organisms living through unfavorable growing conditions during a mass extinction. The term has since been expanded to include a number of patterns such as the preferential survival of smaller taxa (faunal sorting), dwarfing within a single species, and size reduction amongst a variety of clades that includes notable morphological change (miniaturization) (Harries & Knorr, 2009). The Lilliput effect has been reported in a variety of marine invertebrates such as Early Silurian corals (Kaljo, 1996), Late Devonian conodonts (Renaud & Girard, 1999) and Early Danian echinoids (Jeffery, 2001). It has been less documented amongst terrestrial taxa, but decreased body sizes have been seen in land vertebrates across the K-T boundary (Buffetaut, 2006) and amongst the Pleistocene megafaunal extinctions (Brook & Bowman, 2002).

The end-Permian mass extinction (EPME) is widely regarded as the most catastrophic of the five Phanerozoic mass extinctions in Earth’s history (Erwin, Bowring & Yugan, 2002; Irmis & Whiteside, 2012; Benton & Newell, 2014). Current estimations from the marine Global Boundary Stratotype Section and Point at Meishan, China indicate that the main event took place approximately 251.9 million years ago (Ma) (Burgess, Bowring & Shen, 2014). Proposed mechanisms of the extinction include global warming, atmospheric hypoxia (decreased oxygen levels), oceanic anoxia associated with hypercapnia (increased carbon dioxide levels) and/or euxinia (increased sulphide levels) (Wignall & Twitchett, 1996; Knoll et al., 1996; Isozaki, 1997; Hotinski et al., 2001; Kump, Pavlov & Arthur, 2005), excessive methane release (Krull et al., 2000; Sheldon & Retallack, 2002), increased mercury levels (Shen et al., 2019) and regional terrestrial aridification (Ward, Montgomery & Smith, 2000; Ward et al., 2005; Smith & Ward, 2001). Siberian Trap volcanism is currently the most widely proposed driving force behind the extinction, which is thought to have triggered the release of massive amounts of methane, sulphur dioxide and carbon dioxide into the atmosphere (Wignall, 2001; Benton & Twitchett, 2003; Erwin, 2006; Knoll et al., 2007; Algeo et al., 2011; Shen et al., 2011; Payne & Clapham, 2012; Retallack, 2013).

The EPME had a considerable effect on both marine and terrestrial ecosystems, substantially changing community structure, causing a severe reduction in global biodiversity (Xie et al., 2005; Yin et al., 2007; Shen et al., 2011; Chen & Benton, 2012; Smith & Botha-Brink, 2014; Roopnarine & Angielczyk, 2015; Roopnarine et al., 2018; Roopnarine et al., 2019). In the South African Karoo Basin, where the terrestrial aspect of the extinction is best preserved, the post-extinction environment has been described as unpredictable, with increased aridity and temperature extremes (Smith & Botha, 2005; Smith & Botha-Brink, 2014; Rey et al., 2016; MacLeod, Quinton & Bassett, 2017; Botha et al., 2020) (although see Gastaldo et al., 2020 for proposed seasonally wet conditions during the latest Permian). Species survival was variable, with some going extinct (e.g., pareiasaurian parareptiles, gorgonopsian therapsids), others surviving for a time at reduced diversity levels (e.g., dicynodont and therocephalian therapsids) and others radiating after the EPME to become particularly diverse during the Triassic (e.g., temnospondyl amphibians, procolophonid parareptiles, archosauromorph reptiles and cynodont therapsids) (Ruta & Benton, 2008; Nesbitt et al., 2013; Ruta et al., 2013a; Ruta et al., 2013b; MacDougall, Brocklehurst & Fröbisch, 2019). Two recent studies have shown that there is still controversy regarding the position of the terrestrial Permo-Triassic boundary (PTB) in the South African Karoo Basin (Botha et al., 2020; Gastaldo et al., 2020). Based on Gastaldo et al. (2020) the upper Palingkloof Member of the Balfour Formation, which contains the PTB in the main Karoo Basin, is no older than 252.24 ± 0.1 Ma, which would suggest a Permian age. However, it may be as young as 251.7 ± 0.3 Ma based on the identification of even younger detrital zircon populations below this interval (Botha et al., 2020). This most likely places the base of the *Lystrosaurus declivis* Assemblage Zone within the basal Triassic as in the *Lystrosaurus murrayi*-dominated fauna of India’s Panchet beds (Tripathi & Puri, 1962; Tripathi & Satsangi, 1963) consistent with the currently accepted position of the PTB interval in the main Karoo Basin (e.g., Smith & Ward, 2001; Smith & Botha-Brink, 2014; Botha et al., 2020). The placement of the PTB in the Karoo Basin in this study is placed according to Botha et al. (2020) (i.e., the ‘traditional’ placement) and not according to the Gastaldo et al. (2020) alternative higher in the section.

A Lilliput effect across the EPME in the marine realm has been reported for a variety of taxa (Twitchett, 2007) including conodonts (Luo et al., 2008), bivalves, gastropods, brachiopods (Metcalfe, Twitchett & Price-Lloyd, 2011) and foraminifera (Song, Tong & Chen, 2011). Fewer studies have documented such an effect in the terrestrial realm. However, studies on South African therocephalian synapsids have reported post-extinction body size reductions in the form of within-lineage heterochronic shifts (dwarfism in *Moschorhinus kitchingi*) and the differential survival of some small-bodied therocephalians (e.g., baurioids) (Huttenlocker & Botha-Brink, 2013; Huttenlocker & Botha-Brink, 2014). Amongst anomodont dicynodonts large taxa disappeared across the PTB only to reappear later during the Middle Triassic (Angielczyk & Walsh, 2008).

**Figure 1:**
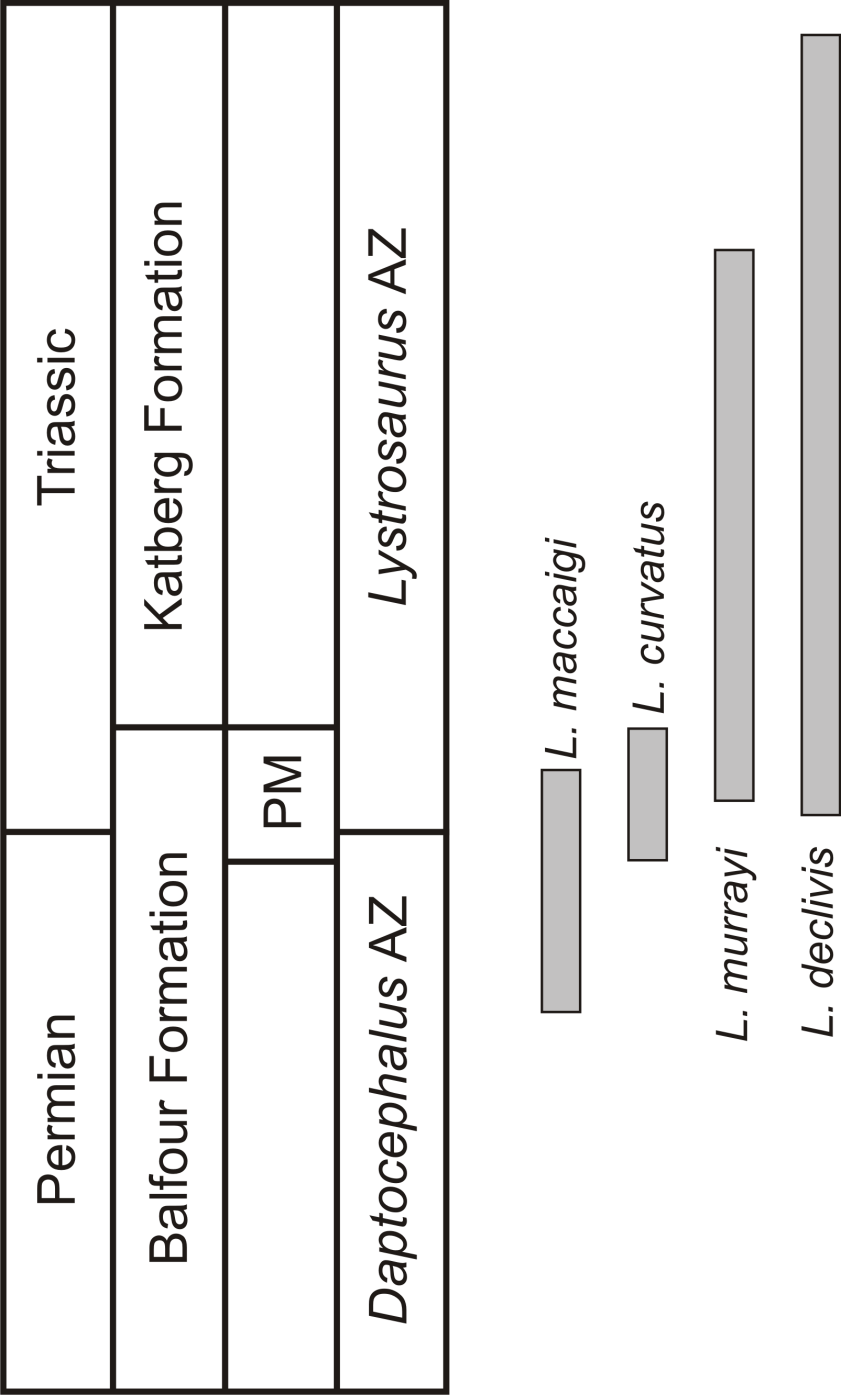
Biostratigraphic ranges of the four South African *Lystrosaurus* species in the Karoo Basin. Abbreviations: AZ, Assemblage Zone; PM, Palingkloof Member.

One of the few tetrapod species that survived the EPME is the well-known dicynodont therapsid *Lystrosaurus*. It is the only dicynodont found on either side of the PTB and constitutes more than 90% of the vertebrate fauna from the Early Triassic *Lystrosaurus declivis* Assemblage Zone (Botha & Smith, 2020). Although relatively rare during the Late Permian, the Early Triassic species inundated post-extinction communities to become the dominant tetrapods in terrestrial ecosystems after the EPME (Smith, Rubidge & Van der Walt, 2012; Botha-Brink, Huttenlocker & Modesto, 2014). In the South African Karoo Basin, the biostratigraphy of the four *Lystrosaurus* species, which comprise *L. maccaigi*, *L. curvatus*, *L. murrayi* and *L. declivis* (Grine et al., 2006; Botha & Smith, 2007), is well-documented (Botha & Smith, 2007; Botha et al., 2020). Until recently, *L. curvatus* was the only South African species to be found on both sides of the PTB (sensu Botha et al., 2020), with *L. maccaigi* disappearing at the PTB and *L. murrayi* and *L. declivis* being found in purely Triassic deposits (Botha & Smith, 2007; Smith & Botha-Brink, 2014). However, a recent study on a relatively new PTB site has revealed that *L. maccaigi* once considered to be solely Permian in South Africa also crossed the boundary (Botha et al., 2020). This is not unexpected as *L. maccaigi* was found in Triassic strata of the Fremouw Formation in Antarctica during the 1980s (Cosgriff, Hammer & Ryan, 1982). Both *L. maccaigi* and *L. curvatus* disappear in the Triassic portion of the Palingkloof Member, Balfour Formation and have yet to be found in the overlying Katberg Formation of the *Lystrosaurus declivis* Assemblage Zone (Botha et al., 2020; Botha & Smith, 2020), whereas the ranges of *L. murrayi* and *L. declivis* continue into the Katberg Formation, with *L. declivis* being found through to the top of the *Lystrosaurus declivis* Assemblage Zone (Botha & Smith, 2006; Botha & Smith, 2020) (Fig. 1). The purely Triassic species, *L. murrayi* and *L. declivis*, are considered to have had smaller body sizes compared to the Permian originating species, *L. maccaigi* and *L. curvatus* (Grine et al., 2006) providing possible evidence for a Lilliput effect in this genus. The excellent biostratigraphical resolution of *Lystrosaurus* makes it possible to assess the presence of such a pattern in this genus, i.e., was there a dwarfing of *Lystrosaurus* during the EPME?

### The history of *Lystrosaurus* osteohistology

An excellent method for assessing life history patterns in extinct animals is the examination of their bone histology or osteohistology. The microstructure of fossil bone records important information about the life history of an extinct vertebrate. These data include ontogenetic, growth, lifestyle and biomechanical information, which would otherwise not be accessible using more traditional methods. The osteohistology of *Lystrosaurus* was first examined by Ricqlès (1972), Ricqlès (1976), and Ricqlès et al. (1991) who examined isolated fragments of humeri and tibiae, as well as Chinsamy & Rubidge (1993) who also briefly assessed a humerus of *L. murrayi*. More recently, Ray, Chinsamy & Bandyopadhyay (2005) provided a comprehensive assessment of the ontogeny of *L. murrayi* (reassessed in Ray, Bandyopadhyay & Bhawal, 2009), and Botha-Brink & Angielczyk (2010) briefly compared the bone tissue patterns of *L. maccaigi*, *L. murrayi* and *L. declivis*. However, none of these studies comprised a comprehensive ontogenetic comparison between the Permian and Triassic species. Botha-Brink & Angielczyk (2010) and more recently Botha-Brink et al. (2016) hypothesized that the high degree of vascularization observed in Triassic *Lystrosaurus* facilitated rapid growth in these species, which may have aided in their survival during this time. However, a more rigorous examination by quantifying Permian and Triassic *Lystrosaurus* life history patterns is required to test this hypothesis. Additionally, Ray, Chinsamy & Bandyopadhyay (2005) provided an alternative hypothesis for its survivorship suggesting that Triassic *Lystrosaurus* was semi-aquatic. This has been a persistent hypothesis since Broom first proposed it in 1902. Consequently, Jasinoski & Chinsamy-Turan (2012) suggested that the abundance of vascular canals was not related to growth rate, but rather increased porosity in Triassic *Lystrosaurus* limb bones. Increased porosity in extant pelagic aquatic mammals has been found to lighten the skeleton and thus, may counteract buoyancy while in the water (Wall, 1983).

As Permo-Triassic *Lystrosaurus* formed such an important part of the ecosystems associated with the EPME and as the lifestyle of this genus remains controversial, the aim of this study is to comprehensively examine changes in body size, bone microanatomy and osteohistology of the four South African *Lystrosaurus* species in order to quantify their life history patterns during the EPME and test the semi-aquatic hypothesis and whether it played a role in *Lystrosaurus* survivorship.

### Methods

Seventy seven limb bones and ribs from 38 individuals of the four South African *Lystrosaurus* species were examined (Table S1). Elements were identified to species level based on the presence of skull material (according to Grine et al., 2006; Botha & Smith, 2007). Limb bones were purposely selected because they document the most complete growth record of the animal. The mid-diaphysis undergoes the least secondary remodeling and thus, elements were thin sectioned in this region wherever possible (Chinsamy, 1990; Chinsamy, 1991; Chinsamy, 1995; Francillon-Vieillot et al., 1990; Horner, de & Padian, 1999). However, as recent studies have found that additional information regarding collagen fibril organisation can be obtained from longitudinal sections and are thus helpful in confirming initial observations from transverse sections, longitudinal sections of the diaphyseal/metaphyseal regions (as the mid-diaphysis was left for transverse sectioning) were also made when possible (Stein & Prondvai, 2014). Elements were only taken from specimens that could be identified to species level.

A database from containing 259 measurements of *Lystrosaurus* basal skull length (BSL) was used to develop a growth distribution for each species (Table S2). For each specimen, the ontogenetic stage or age class was estimated by calculating the BSL as a percentage of the maximum BSL available for the relevant species (389 mm for *L. maccaigi*, 246 mm for *L. curvatus*, 213 mm for *L. murrayi*, and 226 mm for *L. declivis*). Individuals were divided into four age classes based on this database; Age Class I represents juveniles of less than 40% of the maximum known size (MKS) for each particular species; Age Class II represents early subadults of 40–59% MKS; Age Class III represents late subadults of 60–79% MKS and Age Class IV represents adults of 80–100% MKS (after Botha-Brink et al., 2016).

**Figure 2:**
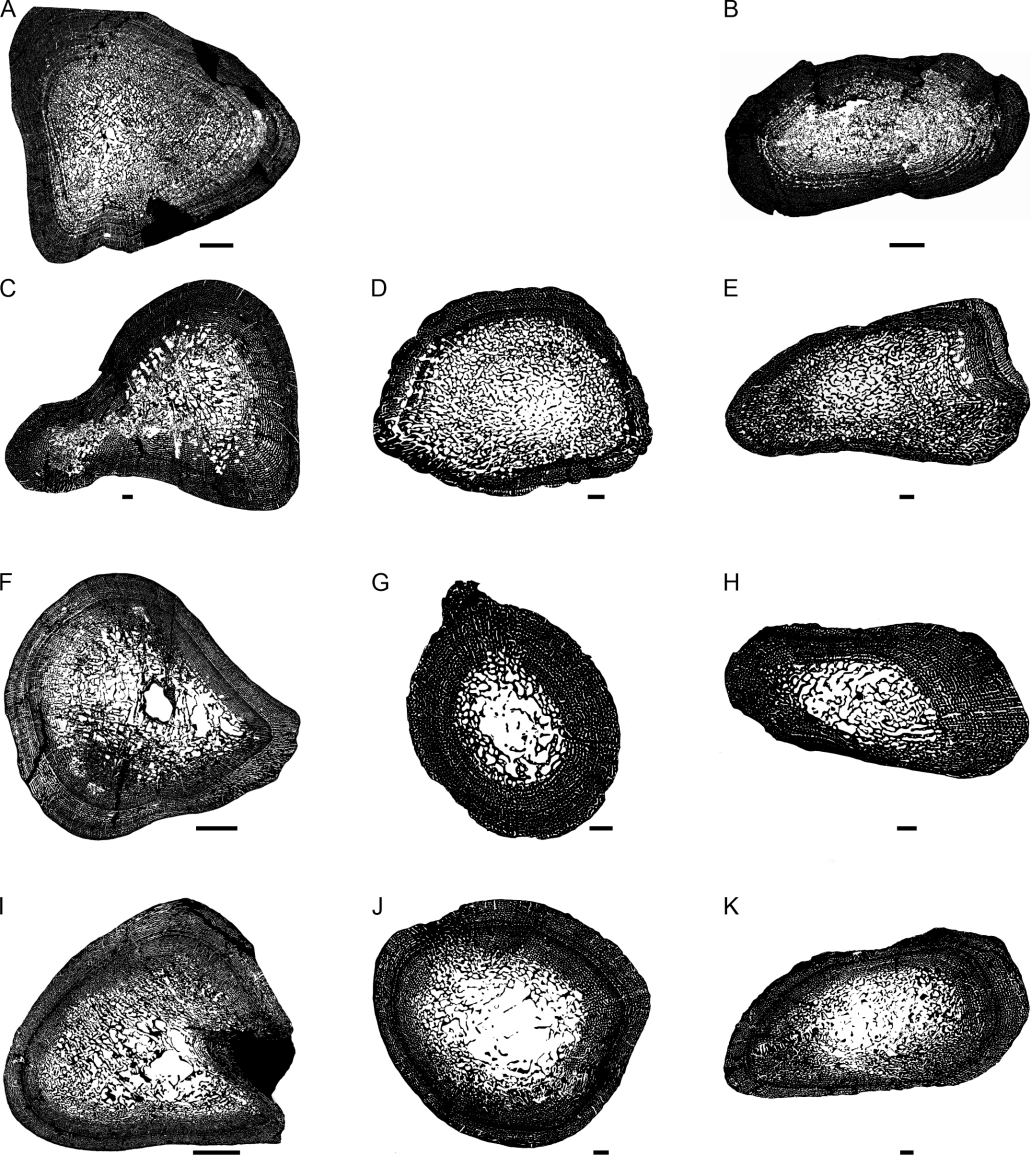
Transverse sections of the largest specimens of the South African *Lystrosaurus* species, used to assess the bone microanatomy. (A) *L. maccaigi* NMQR 3663a humerus. (B) *L. maccaigi* NMQR 3663b ulna. (C) *L. curvatus* NMQR 3922a humerus. (D) *L. curvatus* NMQR 3651b radius. (E) *L. curvatus* NMQR 3651c ulna. (F) *L. murrayi* BP/1/3236 humerus. (G) *L. murrayi* NMQR 659b radius. (H) *L. murrayi* NMQR 659c ulna. (I) *L. declivis* NMQR 1485a humerus. (J) *L. declivis* NMQR 1485b radius. (K) *L. declivis* NMQR 1485c ulna. Scale bars equal 1,000 µm, apart from A, B and I which equal 5,000 µm.

Thin sectioning was conducted at the National Museum, Bloemfontein using standard techniques following that set out in Gower et al. (2014) and Ecker et al. (2015). Each element was embedded in a clear epoxy resin Struers Epofix, to prevent the bones from disintegrating during the thin sectioning process. The embedding was completed within a Struers CitoVac vacuum chamber to remove air bubbles from within the bone and surrounding resin. The resin was left to cure for 24 h and then sequential transverse sections of the midshaft of each element (and when possible longitudinal sections of the diaphyseal/metaphyseal regions), approximately 1.5 mm thick, were cut using a cut-off diamond-tipped saw within a Struers Accutom-50 cut-off grinding machine. Each section was then mounted onto a frosted petrographic glass slide using the resin adhesive LR20, which had been placed in the Struers CitoVac vacuum chamber to remove air bubbles. Pressure was then applied to the sections to eliminate any excess bubbles. Once set, the sections were ground to approximately 60 µm thick (although the thickness varied between elements, due to variable preservation of each bone) using a diamond-tipped grinding wheel within the Struers Accutom-50 cut-off grinding machine. The resulting thin sections were polished until smooth using a Struers LaboPol 5 polishing machine (Gower et al., 2014; Ecker et al., 2015). The bone microstructure was viewed and photographed using a Nikon Eclipse 50i Polarizing microscope and DS-Fi1 digital camera and captured in the computer program NIS Elements D 4.6 (Nikon Corp., Tokyo, Japan). Osteohistology terminology follows that of Francillon-Vieillot et al. (1990), Ricqlès et al. (1991) and Stein & Prondvai (2014).

The thickness of the cortex and the overall compactness of the bone of the largest histologically-sampled individuals of each species were measured in order to assess the robustness of the bones and to make inferences regarding lifestyle (Figs. 2A–2K) as described by Gower et al. (2014). The midshaft cortical thickness (k, expressed from 0 to 1 where cortical thickness is thickest when k tends towards 0, (Currey & Alexander, 1985)) was recorded for the limb bones by measuring the ratio between the inner diameter and outer diameter of the bone in the computer program Bone Profiler for Windows V4.5.8 (Girondot & Laurin, 2003). A bone compactness profile (defined as “the ratio between the surface occupied by bone tissues and the total bone surface”, (Germain & Laurin, 2005:337) was also obtained from the midshaft of each element also using Bone Profiler for Windows. This profile or global compactness (Cg) represents the ratio between the mineralized tissue surface and the total cross-sectional surface and is higher towards a value of 1 (Girondot & Laurin, 2003; Laurin, Girondot & Loth, 2004; Germain & Laurin, 2005; Canoville & Laurin, 2010). Global compactness, Cc (bone center compactness), Cp (bone periphery compactness), Min (minimum compactness), Max (maximum compactness), S (proportional to the width of the transition zone from medullary cavity to cortex) and P (the transition area from medullary cavity to cortex) were used to predict a lifestyle for the humerus (Canoville & Laurin, 2010) and radius (Laurin, Canoville & Germain, 2011). Although there is currently no formula for predicting lifestyle in the ulna, K and Cg were also measured in order to make comparisons between the *Lystrosaurus* species as well as with extant taxa.

The relative vascular area in the primary cortex was measured using the Nikon software NIS elements D 4.6 (Nikon Corp., Tokyo, Japan). As the organization and density of the vascular canals in the primary cortex is closely related to bone deposition rate (e.g., Amprino, 1947; Margerie & Castanet, 2002; Margerie et al., 2004), this method provides a quantitative way for comparing between different elements and taxa. The maximum possible vascularization for each element is quantified because the vascular canals would have included lymph and nerves as well as blood vessels during life (Starck & Chinsamy, 2002). Cortical vascularity was calculated by randomly selecting ten standardized quadrants from the midcortex of each section (thus excluding areas of secondary remodeling). The total canal area was calculated and divided by the total cortical area and then multiplied by 100 to obtain a percentage (Table S1). Mean primary osteon diameter also reflects bone apposition rates and is generally highest in highly woven fibrolamellar bone (Margerie & Castanet, 2002). Mean primary osteon diameter was calculated (in microns) by measuring the transverse widths of primary osteons in the cortex under polarized light (Table S1).

### Results

#### Osteohistology

##### L. maccaigi

**Figure 3:**
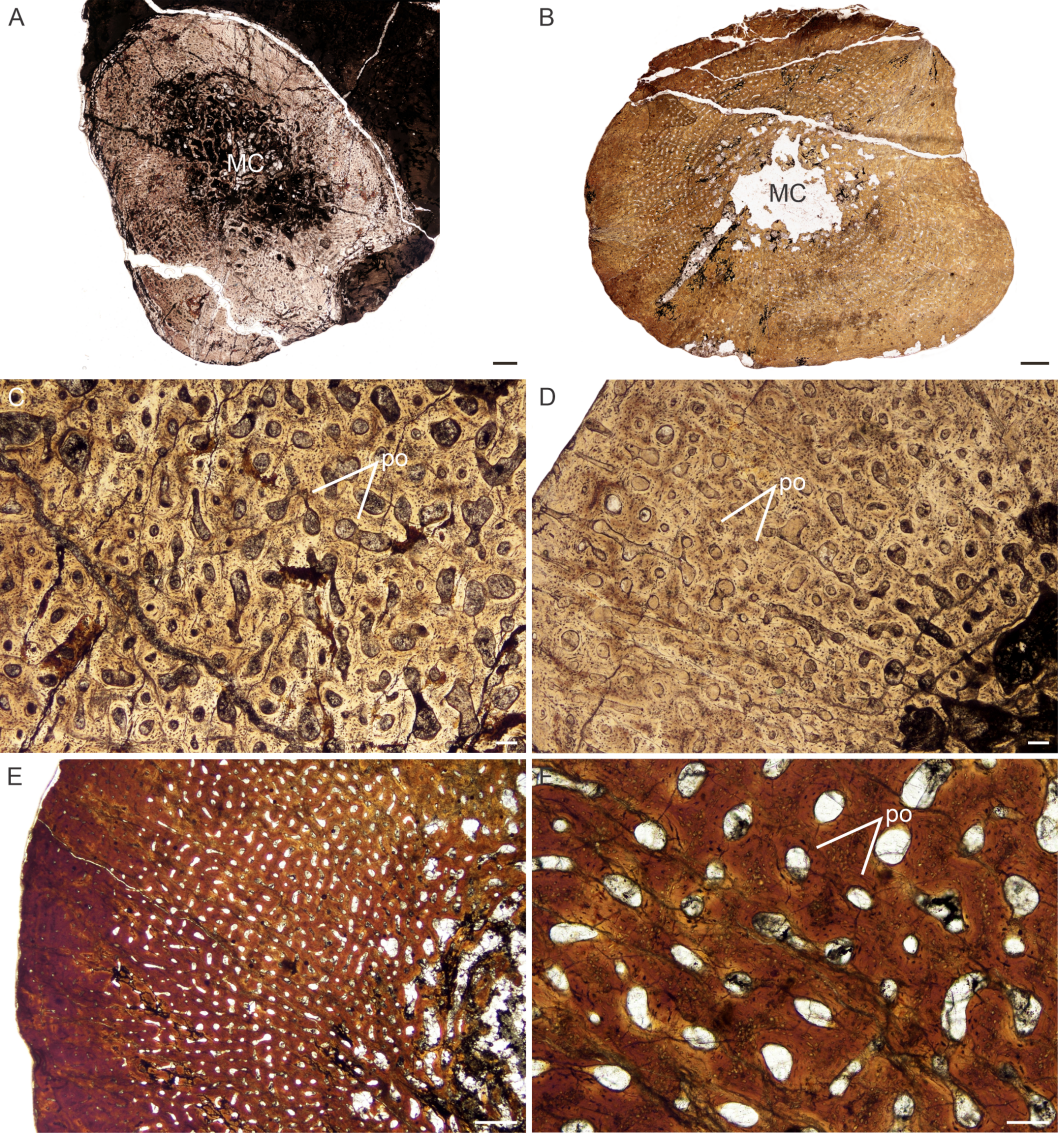
Transverse sections of the bone histology of *Lystrosaurus maccaigi*, Age Class I. (A) NMQR 3658a humerus showing an infilled medullary cavity. (B) NMQR 3641 tibia showing a thick cortex surrounding a small medullary cavity. (C) NMQR 3658a humerus showing woven fibered bone with primary osteons. (D) NMQR 3658b radius showing highly vascularized primary bone tissue. (E) NMQR 3641 tibia showing highly vascularized primary bone tissue. (F) High magnification of NMQR 3641 tibia showing the primary osteons. Abbreviations: MC, medullary cavity; PO, primary osteons. Scale bars equal 100 µm, apart from E, which equals 500 µm and A and B, which equal 1,000 µm.

*Age Class I (<40%)—* Two individuals comprising a humerus, radius (NMQR 3658) and tibia (NMQR 3641) were available for study (Table S1). NMQR 3658 (Fig. 3A) was recovered from Permian deposits whereas NMQR 3641 (Fig. 3B) was recovered from Triassic deposits. Individuals in this ontogenetic stage contain medullary cavities that are infilled by a labyrinth of bony trabeculae (Fig. 3A) or are tiny (Fig. 3B). The perimedullary cancellous bone tissue transforms gradually into a thick compact cortex, which comprises highly vascularized (mean cortical vascularity 13.5%) fibrolamellar bone tissue. The fibrolamellar complex is identified as such by the presence of a woven-fibered interstitial matrix (distinguished by the woven-fibered texture and disorganized non-lamellar nature of the matrix) containing numerous haphazardly-arranged globular osteocyte lacunae and the random arrangement of longitudinally-arranged primary osteons (mean diameter 83 µm) (Figs. 3C–3F). The presence of such bone tissue indicates rapid growth as both woven-fibered bone and high cortical vascularity are linked to rapid bone apposition rates (Amprino, 1947; Margerie & Castanet, 2002; Margerie et al., 2004). The vascular canals decrease in size slightly on one side of the bone in NMQR 3641. Secondary osteons are small and rare, and limited to the peri-medullary region. Growth marks in the form of annuli or Lines of Arrested Growth (LAG) are absent from both individuals (Figs. 3A–3F). These marks indicate a temporary decrease (annuli) or cessation (LAGs) in bone growth and are deposited annually in extant vertebrates (Hutton, 1986). Sharpey’s fibers indicating areas of muscle or tendonous insertion were observed in the deltopectoral crest region of humerus NMQR 3658a, on the lateral side of radius NMQR 3658b as well as the posterior side of tibia NMQR 3641.

**Figure 4:**
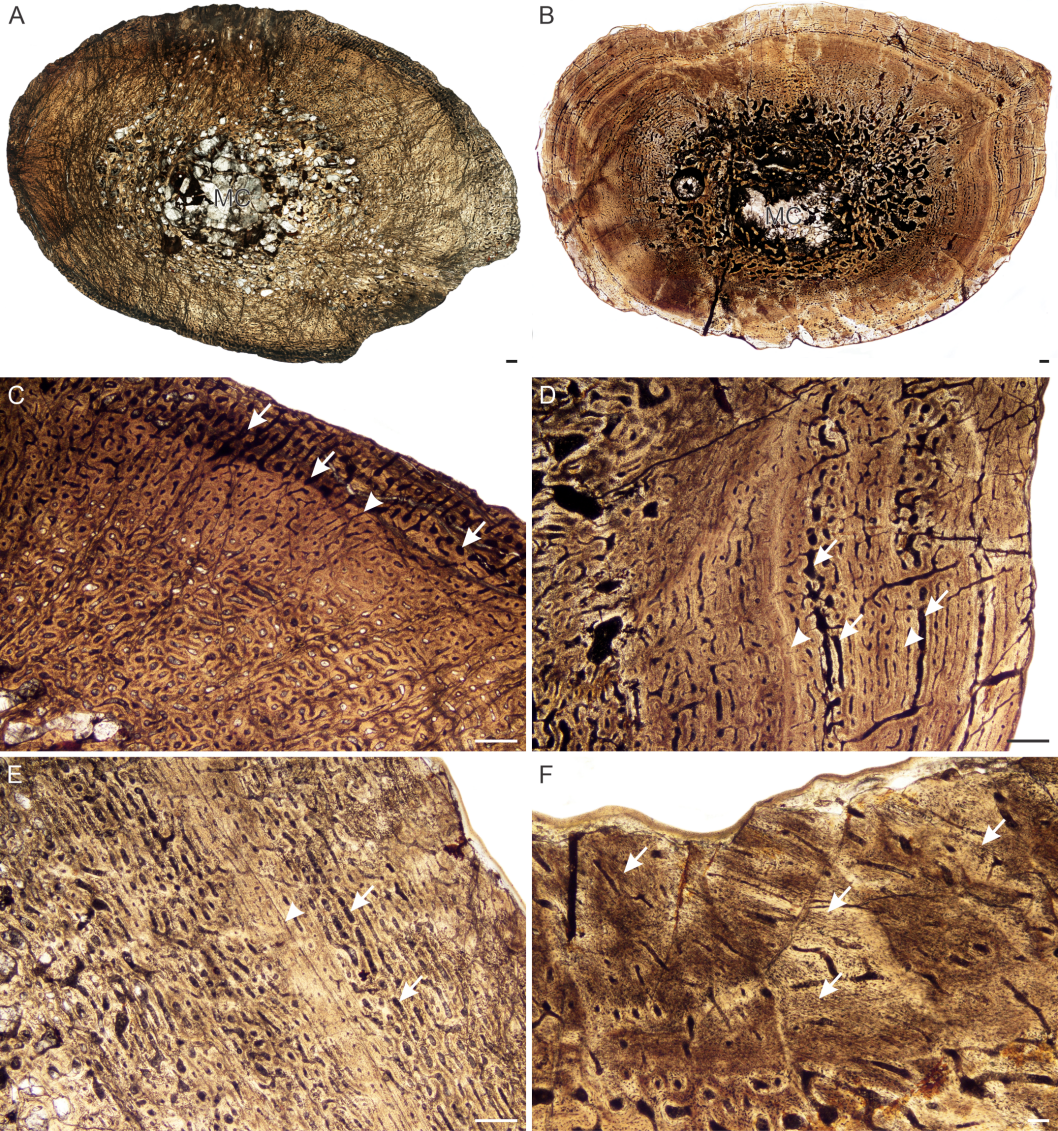
Transverse sections of the bone histology of *Lystrosaurus maccaigi*, Age Class II. (A) NMQR 796a tibia showing an infilled medullary cavity. (B) NMQR 4095 femur showing a gradual transition from medullary cavity to compact cortex. (C) NMQR 796a tibia showing enlarged canals (arrows) and an annulus (arrowhead). (D) NMQR 4095 femur showing enlarged canals (arrows) and two annuli (arrowheads). (E) NMQR 3648 humerus showing large canals (arrows) and a LAG (arrow). (F) NMQR 4095 femur showing Sharpey’s fibers (arrows). Scale bars equal 500 µm, apart from F, which equals 100 µm.

*Age Class II (40%–59%)—* Four individuals including a humerus, femur, tibiae, fibula and rib were available for assessment (Table S1). NMQR 796 (Fig. 4A) and SAM-PK-K10571 were recovered from Permian deposits whereas NMQR 4095 (Fig. 4B) and NMQR 3648 were recovered from Triassic deposits. The medullary cavities are almost completely infilled with trabecular bone, with a few elements having tiny open medullary cavities. The compact cortex is relatively thick. The bone tissues remain similar to those individuals from Stage Class I. However, growth marks in the form of annuli and LAGs are present in all individuals ranging from one to three growth marks (Figs. 4C–4F). In tibia NMQR 796a (47% MKS) a region of relatively small vascular canals is followed by a region of enlarged vascular canals (Fig. 4C). Although a growth mark was not observed separating the two regions, the clear difference suggests some form of cyclicity. I interpret the change in vascular canal size to represent a change in season similar to what a growth mark would represent (Table S1). Similar enlarged vascular canals were found in femur NMQR 4095 (Fig. 4D) and humerus NMQR 3648 (Fig. 4E) associated with annuli. These canals form reticular and laminar networks. They are primary in nature (mean diameter 78 µm) and do not exhibit any characteristics that would suggest they are secondary (e.g., Howship’s lacunae, cement line, overlapping). Secondary osteons are small, isolated and limited to the inner cortex. Sharpey’s fibers were observed in femur NMQR 4095 (Fig. 4F) on the posterior surface possibly for the insertion of M. coccygeo-femoris (Ray, 2006).

**Figure 5:**
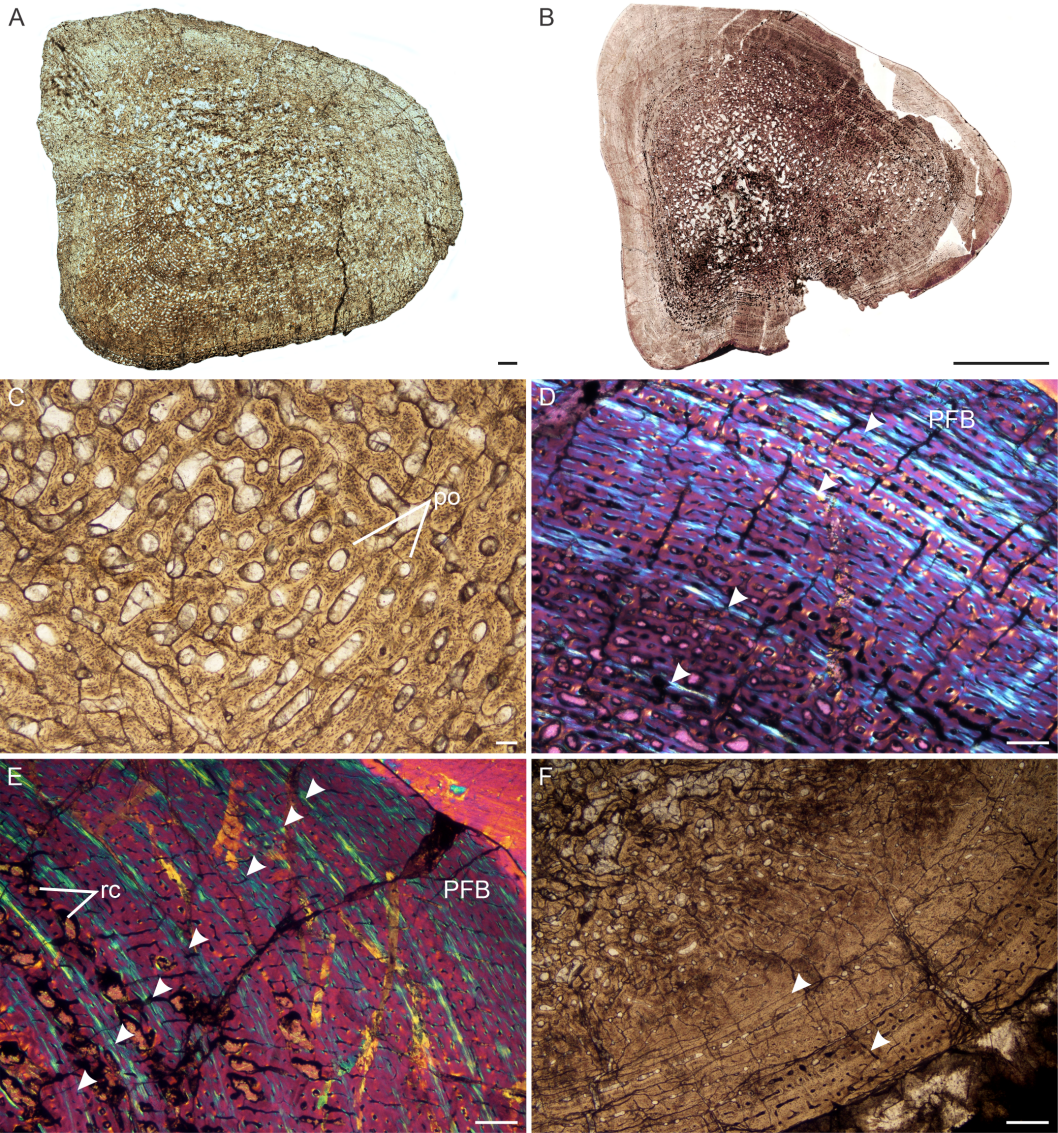
Transverse sections of the bone histology of *Lystrosaurus maccaigi*, Age Class III. (A) NMQR 3689a humerus showing an infilled medullary cavity. (B) NMQR 3663a humerus showing the absence of an open medullary cavity. (C) NMQR 3689a humerus showing highly vascularized primary bone tissue. (D) NMQR 3663 humerus showing annuli (arrowheads) and peripheral parallel-fibered bone tissue. (E) NMQR 3663 ulna showing numerous annuli (arrowheads) and peripheral parallel-fibered bone tissue. (F) NMQR 3689c tibia showing two LAGs (arrowheads). Abbreviations: PFB, parallel-fibered bone; PO, primary osteons; RC, resorption cavities. Scale bars equal 500 µm, apart from A, B , which equal 1,000 µm and C, which equals 100 µm.

*Age Class III (60%–79%)—* Four individuals including humeri, femora and a tibia were available for study (Figs. 5A–5F). One individual, NMQR 3689, was recovered from Triassic deposits, the rest are from Permian deposits (Table S1). The medullary cavities are almost completely infilled with a generally gradual transition zone from cavity to compact cortex (Figs. 2A, Figs. 2B, 5A, 5B). The cortex continues to be dominated by fibrolamellar bone with vascular canals in a laminar longitudinally-oriented network. The smallest individual in this age class, NMQR 3689 (Fig. 5C), is highly vascularized (mean cortical vascularity 16%, Table S1) with large primary vascular canals (mean diameter 78 µm, Table S1). The largest elements have slightly smaller vascular canals (mean primary osteon diameter 71 µm, Table S1) and the bone tissue becomes parallel-fibered from the mid-cortex (e.g., NMQR 3663, Fig. 5D). Secondary osteons are rare in NMQR 3689, but become increasingly prevalent in the larger elements. Secondary resorption is extensive, with resorption cavities extending into the mid-cortex in places (Fig. 5E).

It is difficult to discern any growth marks in the NMQR 3689a humerus, but three annuli are present in the femur and tibia of this individual (Fig. 5F). Growth marks are present in the rest of the elements as well and consist of annuli of lamellar bone or LAGs (Fig. 5E). Sharpey’s fibers were observed in femur NMQR 3699a on the anterior surface, possibly for the insertion of M. adductor femoris (Ray, 2006). A region on the posteromedial side of ulna NMQR 3663b consists of collagen fibers and vascular canals that face perpendicularly to the rest of the bone tissues and may represent an area of muscle insertion.

**Figure 6:**
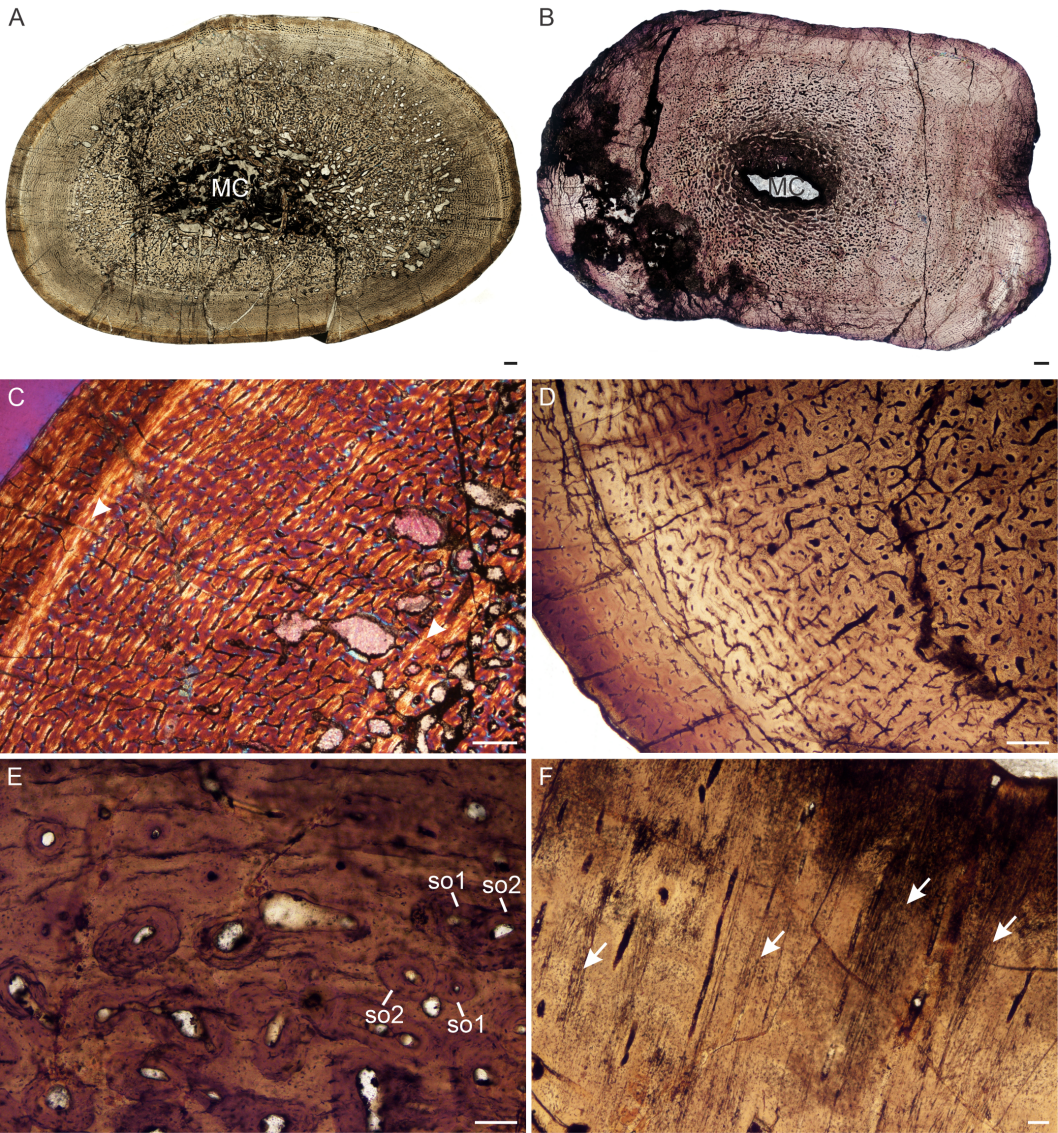
Transverse sections of the bone histology of *Lystrosaurus maccaigi*, Age Class IV. (A) NMQR 1020 femur showing an infilled medullary cavity. (B) NMQR 3940b tibia showing a tiny medullary cavity. (C) NMQR 1020 femur in cross-polarized light showing an annulus (arrowhead). (D) NMQR 3940b showing relatively smaller vascular canals towards the sub-periosteal surface. (E) NMQR 3940d radius showing two generations of secondary osteons. (F) NMQR 3940d radius showing Sharpey’s fibers (arrows). Abbreviations: MC, medullary cavity; SO, secondary osteon. Scale bars in A and B equal 1,000 µm, C and D equal 500 µm, and E and F equal 100 µm.

*Age Class IV (80%–100%)—* Two individuals, both from Permian deposits, were available for study (Table S1). The bones contain small medullary cavities with an extensive spongy region surrounding the cavities (Figs. 6A, 6B). The bone tissues in both individuals consist of fibrolamellar bone, but it becomes parallel-fibered in tibia NMQR 3940b and radius NMQR 3940d. Femur NMQR 1020 contains fibrolamellar bone to the sub-periosteal surface and there is no indication of a decrease in growth rate (Fig. 6C). This individual also exhibits fewer growth marks than humerus NMQR 3663 from Age Class III, with NMQR 3663 being only 3% smaller than NMQR 1020 (Table S1). This difference may be due to intraskeletal or inter-individual variability. NMQR 3940 shows a decreased size in the vascular canals, which become increasingly simple towards the bone periphery (Fig. 6D). Outer circumferential lamellae or OCL (also known as an External Fundamental System) in the form of avascular lamellar bone and or many closely spaced LAGs at the bone periphery, which would indicate that maximum growth had been reached, are absent. An OCL has been observed in the Triassic kannemeyeriiform dicynodonts *Kannemeyeria* and *Placerias* (Botha-Brink & Angielczyk, 2010; Green et al., 2010). Secondary osteons in this individual are more abundant and extend to the sub-periosteal surface in places and have probably resorbed some of the inner growth marks. Up to two generations of secondary osteons were observed (Fig. 6E). Sharpey’s fibers were observed in radius NMQR 3940D (Fig. 6F).

##### L. curvatus

Only specimens belonging to Age Classes I and III were available for study. This is because *L. curvatus* is a rare species with a relatively short stratigraphical range.

**Figure 7:**
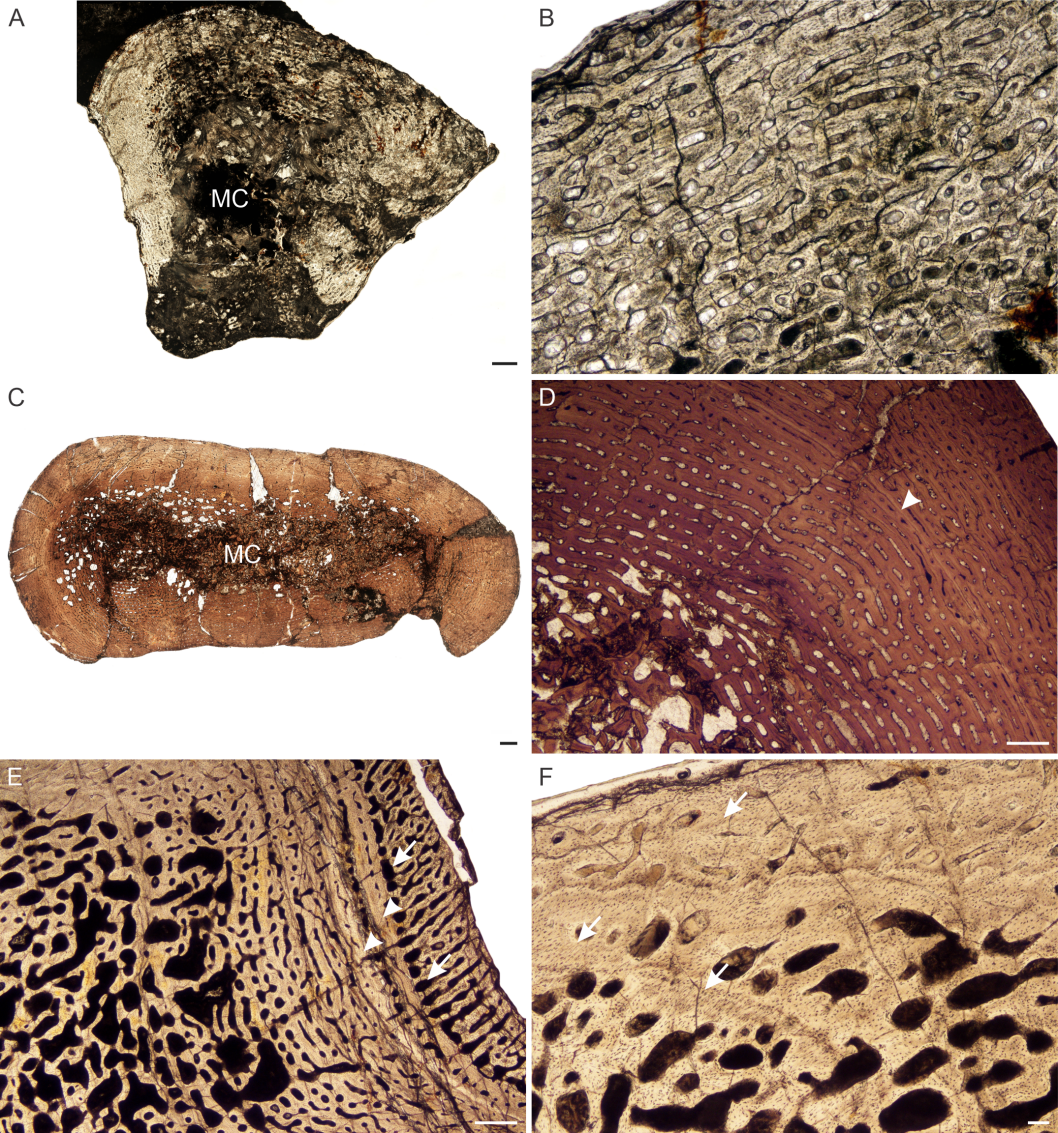
Transverse sections of the bone histology of *Lystrosaurus curvatus*, Age Class I, III. (A) NMQR 4001 humerus showing a tiny medullary cavity and thick cortex. (B) NMQR 4001 humerus (Age Class I) showing highly vascularized fibrolamellar bone tissue. (C) NMQR 3922b femur showing bony trabeculae filling the medullary cavity. (D) NMQR 3922a humerus (Age Class III) showing fibrolamellar bone in a laminar vascular arrangement interrupted by a LAG (arrowhead). (E) NMQR 3651c ulna (Age Class III) showing enlarged canals (arrows) following two annuli (arrowheads). (F) NMQR 3651b radius (Age Class III) showing Sharpey’s fibers (arrows). Abbreviations: MC, medullary cavity; PO, primary osteons. Scale bars in A and B equal 1,000 µm, C and F equal 100 µm, whereas D and E equal 500 µm.

*Age Class I (<40%)—* One individual, NMQR 4001, comprising a humerus, recovered from Triassic deposits, was available for study (Table S1). The medullary cavity is relatively small and open (Fig. 7A). Secondary reconstruction is absent. The bone tissues are similar to those of *L. maccaigi* for this age class. They consist of highly vascularized fibrolamellar bone with large (primary osteon diameter 80 µm, Table S1), laminar or longitudinally-oriented primary vascular canals (Fig. 7B). There is no decrease in vascularization towards the periphery and growth marks are absent.

*Age Class III (60%–79%)—* Two individuals comprising a radius, ulna, humeri and femur, both from Permian deposits, were available for examination (Table S1). The medullary cavities are infilled with bony trabeculae (Fig. 7C) resulting in a gradual transition zone from cavity to compact cortex (Figs. 2C–2E). The bone tissues continue to contain highly vascularized fibrolamellar bone in a laminar network (Fig. 7D). The size of the vascular canals decreases towards the sub-periosteal surface. Growth marks are present as faint annuli in all the bones. In individual NMQR 3651, which comprises a humerus, radius and ulna, the vascular canals decrease in size towards the annulus and increase in size again after the annulus, showing an increase in growth rate after this growth mark (Fig. 7E). An OCL was not found. Sharpey’s fibers were observed in radius NMQR 3651b on the anterolateral side and in ulna NMQR 3651c on the medial side (Fig. 7F).

##### L. murrayi

**Figure 8:**
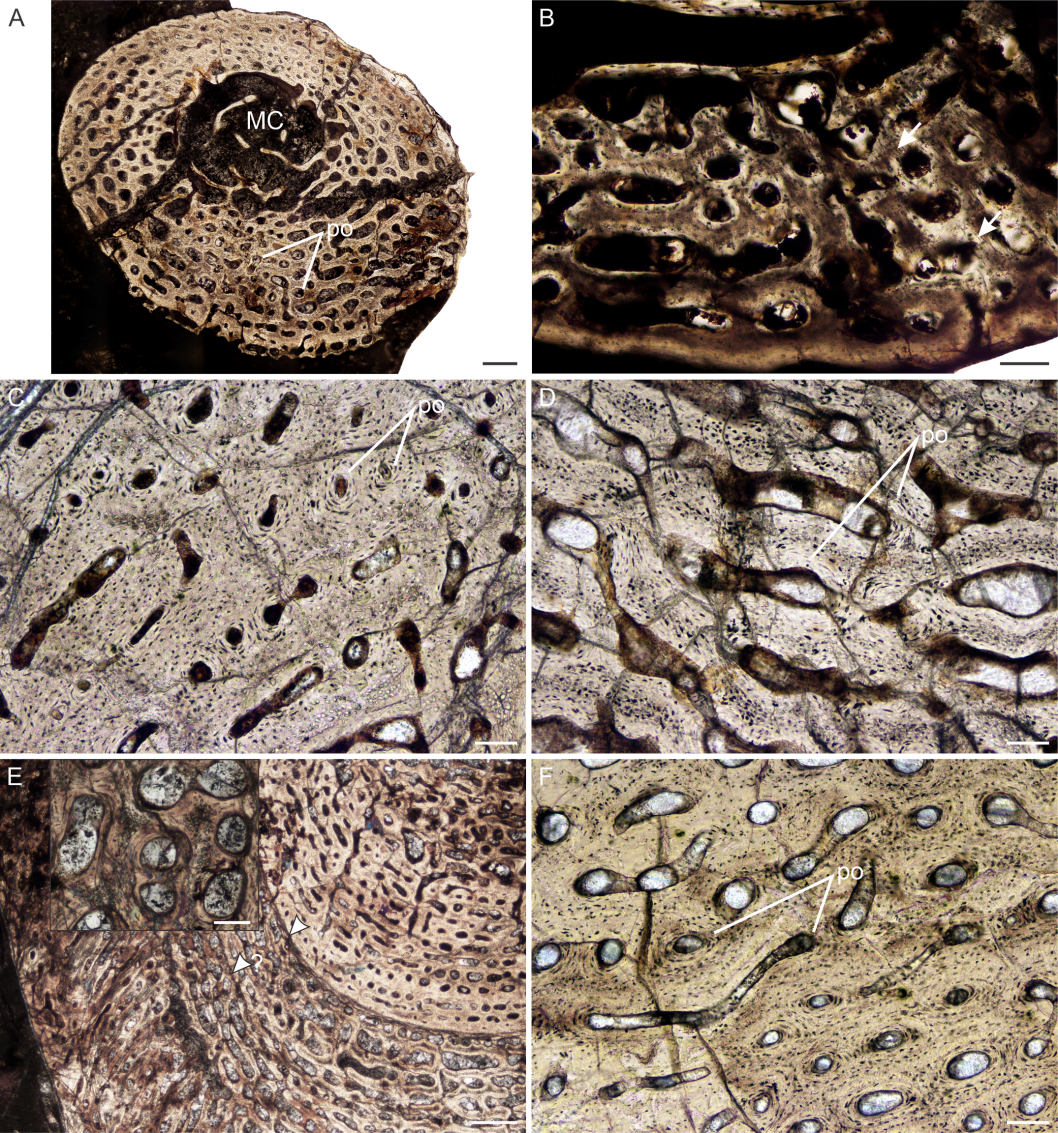
Transverse sections of the bone histology of *Lystrosaurus murrayi*, Age Class I–IV. (A) NMQR 3635a radius (Age Class I) showing highly vascularized woven-fibered bone tissue. (B) NMQR 3635b ulna (Age Class I) showing Sharpey’s fibers (arrows). (C) NMQR 3927a humerus (Age Class II) showing highly vascularized primary bone tissue. (D) High magnification of humerus BP/1/5070 (Age Class III) showing primary osteons. (E) SAM-PK-8991 (Age Class III) showing a change from laminar to radial vascular canals traversed by two annuli (arrowheads). High magnification insert shows the secondarily enlarged canals. (F) BP/1/3236 humerus (Age Class IV) showing primary bone. Abbreviations: MC, medullary cavity, PO, primary osteons. Scale bars in A and E equal 500 µm, whereas B, C, D and F and insert equal 100 µm.

*Age Class I (<40%)—* All *L. murrayi* individuals were recovered from Triassic deposits. Three individuals comprising humeri, radii and ulnae fall into this age class (Table S1). The smallest individual, NMQR 3635, is 22% the maximum known size and represents the smallest individual of the four *Lystrosaurus* species. The medullary cavities of the radius and ulna of this individual are small, but open, with the radius containing one or two loose trabeculae in the cavity. The cortices are relatively thick. The bone tissues are comprised of fibrolamellar bone with large vascular canals arranged longitudinally in circumferential rows around the medullary cavities (Fig. 8A). Secondary reconstruction and growth marks are absent. Sharpey’s fibers were observed in the ulna (Fig. 8B). The larger bones in this age class contain similar tissues, but the medullary cavities are increasingly infilled by bony trabeculae.

*Age Class II (40%–59%)—* The three individuals (Table S1) comprising humeri, a radius, ulna and femur, contain similar bone tissues to those of Age Class I, i.e., highly vascularized fibrolamellar bone (Fig. 8C). The primary osteons are arranged either in a laminar network or longitudinally in circumferential rows. Resorption cavities and secondary osteons are absent. Growth marks were not observed in any of these bones.

*Age Class III (60%–79%)—* Although the bone tissues of these six individuals (comprising all six limb bones) contain similar bone tissues to the earlier age classes (Figs. 8D; 2G, 2H), some of them exhibit growth marks. The humerus of SAM-PK-8991 for example exhibits two growth marks, whereas others in this age class exhibit one or none. The second growth mark in SAM-PK-8991, however, is not clearly defined, but instead is demarcated by a change in the orientation of vascular canals from laminar to radial (Fig. 8E). This observation does not represent a temporary decrease or cessation in bone growth, but possibly a change in season where the bone growth was accelerated (as expressed by the radial canals). However, it should be noted that this condition of radial canals was only observed in the humerus of SAM-PK-8991 and not in the radius and ulna. The canals before the first growth mark in humerus SAM-PK-8991 appear to be primary, but they appear secondarily enlarged after this first growth mark (Fig. 8E). The canals continue in this manner right up to the periphery of the bone. The cortical porosity is also the highest in the *L. murrayi* sample, even compared to ontogenetically younger individuals. These features were not found in any other specimen and may represent some form of pathology. The ulna and radius of this specimen are very poorly preserved with numerous cracks and thus, it is difficult to determine if the osteons in the mid and outer cortex are primary or secondary.

**Figure 9:**
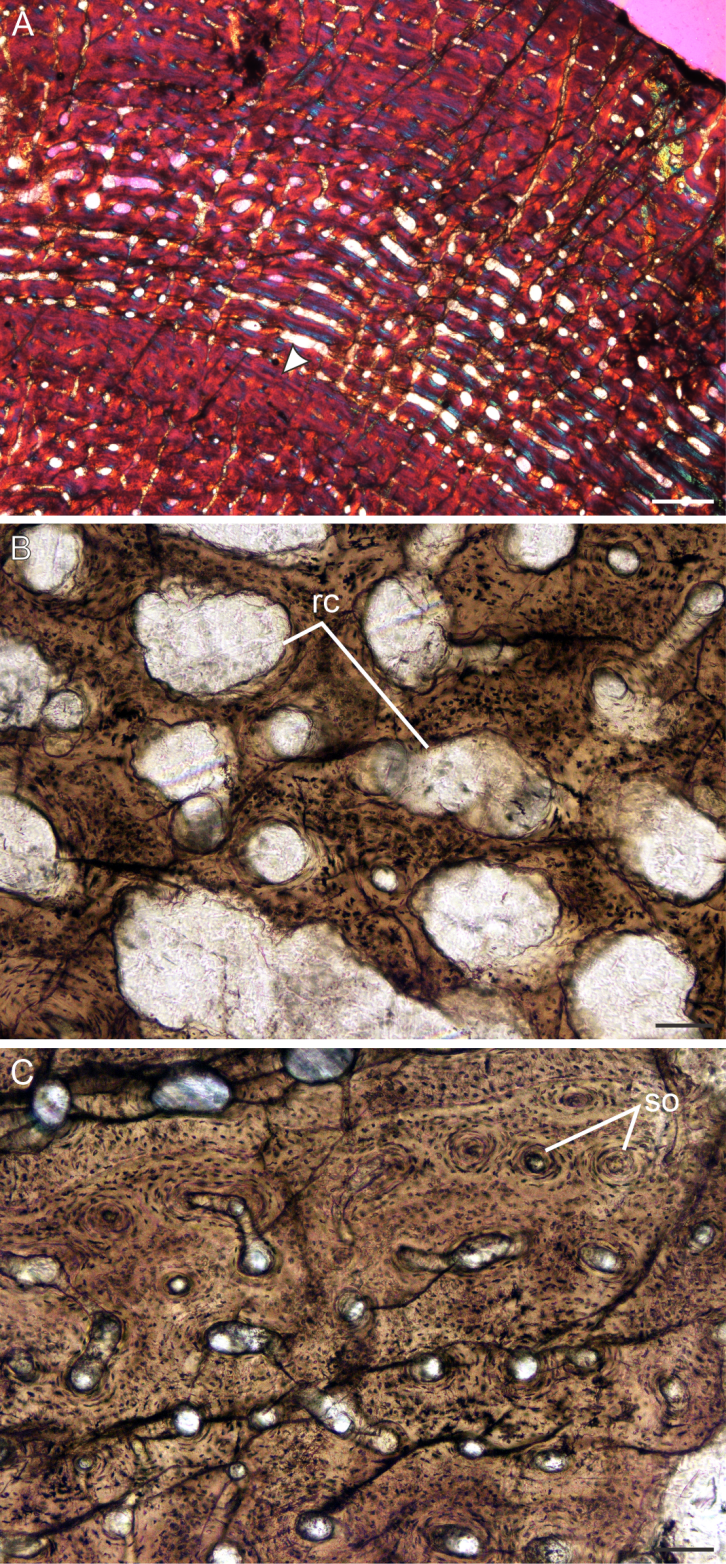
Transverse sections of the bone histology of *Lystrosaurus murrayi*, Age Class IV. (A) BP/1/3236 humerus in cross polarized light showing an annulus traversing fibrolamellar bone tissue. (B) BP/1/3236 humerus showing resorption cavities. (C) BP/1/3236 humerus showing secondary osteons. Abbreviations: RC, resorption cavities, SO, secondary osteons. Scale bars in A equal 500 µm, whereas B and C equal 100 µm.

*Age Class IV (80%–100%)—* Humerus BP/1/3236 is 100% the maximum known size for *L. murrayi*. A very small clear medullary cavity is surrounded by a large expanse of trabecular bone (Fig. 2F). The compact cortex consists primarily of highly vascularized fibrolamellar bone tissue (Fig. 8F). The vascular canals are arranged in a laminar network. They decrease in size towards a mid-cortical annulus and then increase in size again after it (Fig. 9A). Only one growth mark was observed. There is a slight decrease in vascular size at the sub-periosteal surface in places, but slower forming bone tissues are absent. Resorption cavities extend into the mid-cortex in some regions. They are identified by their enlarged appearance and uneven borders in the form of Howship’s lacunae (Fig. 9B). Isolated secondary osteons were observed, scattered throughout the cortex, but dense Haversian bone is absent (Fig. 9C). An OCL was not observed. There is also no indication of a transformation in the cortex from fibrolamellar bone to slower forming bone tissues such as parallel-fibered or lamellar bone.

##### L. declivis

There is no Age Class I for this species. The smallest individual is 44% the maximum known size.

**Figure 10:**
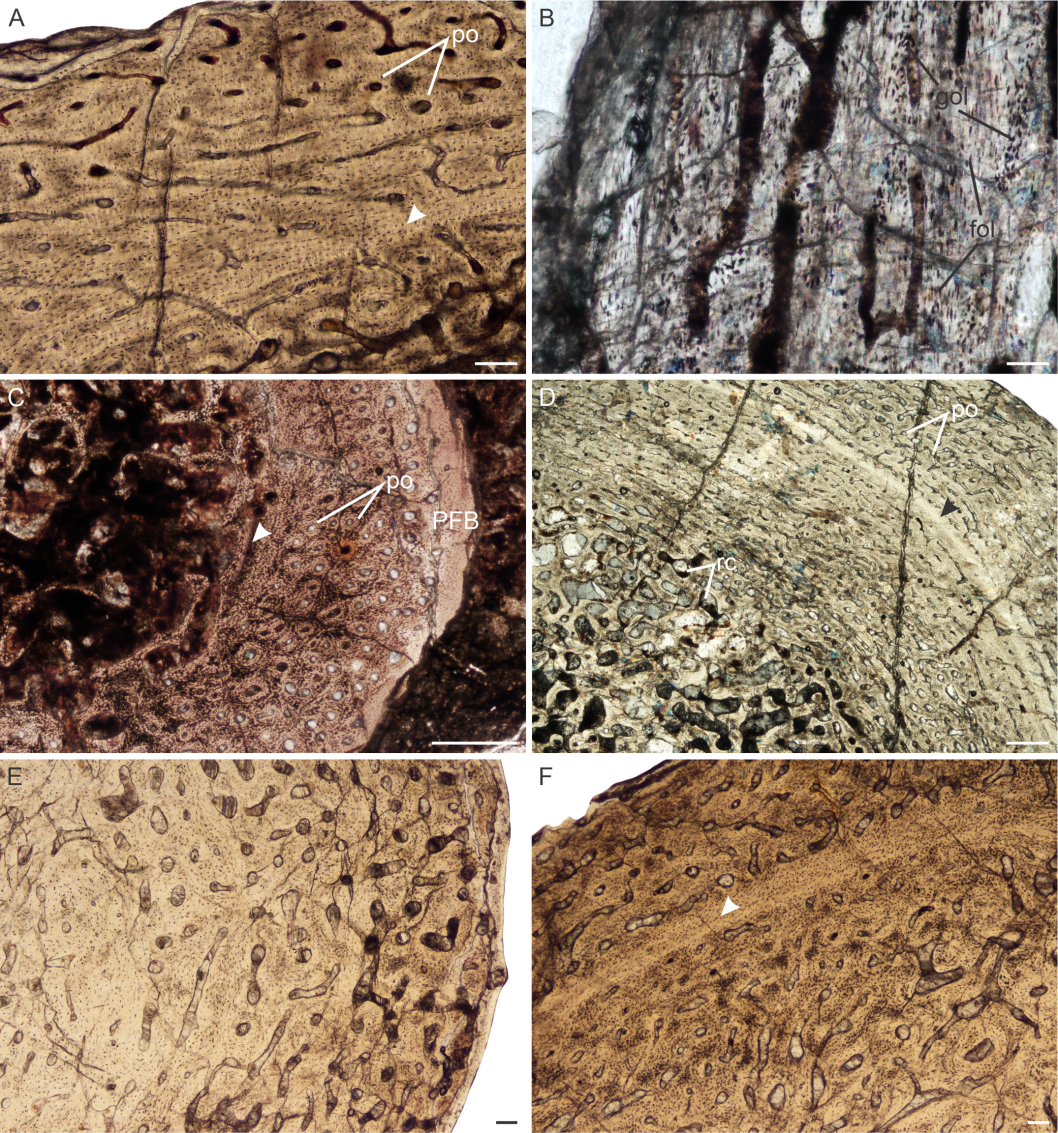
Bone histology of *Lystrosaurusdeclivis*, Age Class II–IV. (A) Transverse section of NMQR 4039 femur (Age Class II) showing fibrolamellar bone tissue traversed by an annulus. (B) Longitudinal section of NMQR 3181 humerus (Age Class III) showing a mixture of globular and flattened osteocyte lacunae. The large, globular osteocyte lacunae indicate the presence of woven-fibered bone. (C) Transverse section of SAM-PK-K8013a rib (Age Class III) showing primary osteons and peripheral parallel-fibered bone. Arrowhead indicates an annulus. (D) Transverse section of NMQR 1485a humerus (Age Class IV) showing an inner region of resorption cavities and mid- to outer region of fibrolamellar bone. Arrowhead indicates an annulus. (E) Transverse sections of NMQR 1485b radius (Age Class IV) and (F) NMQR 1485c ulna (Age Class IV) showing no decrease in vascularization towards the sub-periosteal surface. Arrow in (F) indicates an annulus. Abbreviations: FOL, flattened osteocyte lacunae; GOL, globular osteocyte lacunae; PFB, parallel-fibered bone tissue; PO, primary osteons; RC, resorption cavities. Scale bars in A and B equal 100 µm, whereas C–F equal 500 µm.

*Age Class II (40%–59%)—* All *L. declivis* specimens were recovered from Triassic deposits. Five individuals comprising all six limb bones were available for study (Table S1). Most of the bones contain very small open medullary cavities, which are then surrounded by a wide region of spongy bone. These bone trabeculae in the peri-medullary region result in a broad transition zone to the compact cortex. The bone tissues are similar to those of *L. murrayi* for this stage, which comprise highly vascularized fibrolamellar bone tissue (Fig. 10A). Some individuals exhibit a single mid-cortical annulus (Fig. 10A), but not all limb bones of an individual present growth marks, nor are their presence linked to size. Resorption cavities are limited to the peri-medullary region, as are small isolated secondary osteons.

*Age Class III (60%–79%)—* Four individuals comprising humeri, a radius, ulna, femur and ribs fall into this ontogenetic stage (Table S1). Highly vascularized fibrolamellar bone continues to dominate the cortex. Longitudinal sections of humerus NMQR 3181 (77% of the maximum known size) were made and found to preserve an interstitial matrix of disorganized collagen fibers and densely packed randomly distributed globular osteocyte lacunae, confirming the presence of woven-fibered bone even in relatively older individuals belonging to Age Class III (Fig. 10B). A small region of peripheral parallel-fibered bone was observed in rib SAM-PK-K8013a (Fig. 10C), similar to what was described in Ray, Chinsamy & Bandyopadhyay (2005). Annuli were not observed in the smaller bones of this age class apart from a single annulus in rib SAM-PK-11184a. Single annuli were found in the limb bones and ribs of the slightly larger bones (SAM-PK-11184a, SAM-PK-K8013a, NMQR 3181), however. Resorption cavities are present in the peri-medullary region and extend into the mid-cortex in places. Secondary osteons remain isolated and limited to the inner regions of the bones.

*Stage Class IV (80%–100%)—* A single individual, NMQR 1485, comprising a humerus, radius, ulna and femur represents this ontogenetic stage and is 82% the maximum known size of *L. declivis* (Table S1). The humerus contains a tiny open medullary cavity that is surrounded by an extensive network of bony trabeculae. The other limb bones contain a few loose trabeculae throughout the medullary cavities to some extent (Figs. 2I–2K). The primary bone tissue comprises well vascularized fibrolamellar bone (Fig. 10D), with the humerus and femur having the highest vascularization (Table S1). A single mid-cortical annulus was observed in each limb bone. The ulna may contain two annuli, but the innermost one does not extend around the entire cortex and joins up with the outer one in one area and thus, this inner mark cannot be confirmed as a separate seasonal growth mark. Although there is a slight decrease in the size of some of the vascular canals at the sub-periosteal surface, there is no transition to slower forming tissues and outer circumferential lamellae are absent from the bones indicating that this individual was still actively growing at the time of death. Resorption cavities extend into the mid-cortex and secondary osteons are found scattered through the bones, but dense Haversian bone is absent. Sharpey’s fibers were observed on the anterolateral side of radius NMQR 1485b.

##### Bone microanatomy

The bone microanatomy of the largest, ontogenetically oldest specimens were examined in order to compare between species (Fig. 2). Thus, only the forelimb bones were studied. The cortical thickness (K in Table 1) ranges from 0.38 to 0.69. The radius and ulna cortices of *L. murrayi* and *L. declivis*, and the ulna of *L. maccaigi* are thick (0.38 to 0.51), whereas the radius and ulna of *L. curvatus* are relatively thinner (radius 0.69; ulna 0.67). The cortical thickness of the *L. murrayi* and *L. declivis* humeri are also relatively thicker compared to *L. maccaigi* and *L. curvatus* (Table 1). However, none of the values compare with the closely related dicynodontoid *Daptocephalus leoniceps*, which has a very thick cortex with a *K* value of 0.15.

**Table 1:** Compactness parameters, K and lifestyle readings for the humerus, radius and ulna of the four South African *Lystrosaurus* species, compared to the dicynodontoid *Daptocephalus leoniceps*.

Species	Acc. No.	MD	Min	Max	S	P	Cc	Cp	Cg	K	Lifestyle
**Humerus**											
*L. maccaigi*	NMQR 3663a	40	0.3730496	0.999999	0.2038591	0.6227462	0.399	0.913	0.717	0.62	0
*L. curvatus*	NMQR 3922a	29	0.3879353	0.9234601	0.0748487	0.6072952	0.474	0.931	0.715	0.61	0
*L. murrayi*	BP/1/3236	30	0.2971604	0.85865	0.118658	0.4562598	0.334	0.825	0.716	0.45	0
*L. declivis*	NMQR 1485a	28	0.2884796	0.8827366	0.1255311	0.5596468	0.296	0.865	0.669	0.55	0
*D. leoniceps*	NMQR 3633a	39	0.1910163	0.985676	0.4026052	0.1560593	0.517	0.898	0.798	0.15	0
**Radius**											
*L. curvatus*	NMQR 3651b	18	0.2316513	0.999999	0.1180121	0.6902793	–	–	0.61	0.69	0
*L. murrayi*	NMQR 659b	12	0.1359149	0.9222332	0.0856374	0.387408	–	–	0.783	0.38	0
*L. declivis*	NMQR 1485c	18	0.0857202	0.9095449	0.0772092	0.5071929	–	–	0.679	0.51	0
**Ulna**											
*L. maccaigi*	NMQR 3663b	41	0.3048958	0.999999	0.1806802	0.497793	–	–	0.772	0.49	–
*L. curvatus*	NMQR 3651c	20	0.4636301	0.9041376	0.1178336	0.6704773	–	–	0.691	0.67	–
*L. murrayi*	NMQR 659c	19	0.3233334	0.9169428	0.083876	0.4531913	–	–	0.78	0.45	–
*L. declivis*	NMQR 1485c	23	0.0650608	0.9073332	0.1125244	0.4720466	–	–	0.684	0.47	–

**Notes.**

Abbreviations Acc. No accession number Cc compactness at the bone center Cg global compactness Cp compactness at the bone periphery Max maximum compactness MD maximum diameter Min minimum compactness K cortical thickness P transition area between the medullary cavity and compact cortex S value proportional to the width of the transition zone between the medullary cavity and cortex

A 0 lifestyle reading for the humerus and radius in all taxa indicates an aquatic lifestyle (Canoville & Laurin, 2010; Laurin, Canoville & Germain, 2011). There is currently no available model to determine lifestyle using the ulna.

The overall bone compactness (Cg) is similar among the humeri, with *L. declivis* having a slightly lower compactness (Table 1). Both the *L. murrayi* radius and ulna have slightly higher global compactness values than the other *Lystrosaurus* species. The *D. leoniceps* humerus has the highest Cg, but is only slightly higher than the *L. maccaigi*, *L. curvatus* and *L. murrayi* humeri, all of which are similar (0.715–0.717).

The maximum diameter (MD), Min, Max, S, P, Cc, Cp and Cg values were used to predict the lifestyle of the *Lystrosaurus* species and *D. leoniceps* using Bone Profiler for Windows (Table 1). Only the radius and humerus were used to infer lifestyle as there is currently no model available for the ulna. An aquatic lifestyle was predicted for all the *Lystrosaurus* species using either the humerus or radius as well as for the *D. leoniceps* humerus.

### Discussion

The predominant bone tissue type in *Lystrosaurus* is fibrolamellar bone, as noted in previous studies (Ray, Chinsamy & Bandyopadhyay, 2005; Botha-Brink & Angielczyk, 2010; Botha-Brink et al., 2016). Highly vascularized fibrolamellar bone with longitudinally-oriented vascular canals characterize the juvenile age class, which becomes increasingly laminar through ontogeny. Incipient fibrolamellar bone (a mixture of parallel-fibered bone and woven-fibered bone, Klein, 2010) as described by Olivier et al. (2017) in some *Lystrosaurus* elements is only present in older individuals where patches of parallel-fibered bone have appeared between the fibrolamellar bone. Fully developed fibrolamellar bone is present in the juveniles and early subadults.

There is no dominance of secondary osteons or dense Haversian bone in the mid to outer cortex of any of the individuals studied. Erosionally enlarged channels or resorption cavities are limited to the inner cortex and only extend into the mid-cortex in the largest individuals (Age Class IV). Ray, Chinsamy & Bandyopadhyay (2005) identified erosionally enlarged channels and profuse secondary osteons in the inner to mid-cortex in their subadult individuals and even during early ontogeny in some cases (e.g., femora 40–50% of their adult size) resulting in a very thin compact cortical region. The size of the primary osteons (measured as the maximum diameter of the entire osteon, Table S1) in the mid-cortical regions of the bones examined in this study is consistent with that of other Permo-Triassic therapsids (see Table S1 of Botha-Brink et al., 2016 for other therapsid measurements). The largest *L. murrayi* and *L. declivis* individuals do, however, exhibit bands of small channels that either precede or surround a growth mark that are then followed by bands of relatively larger channels. These larger channels appear to be primary with no secondary enlargement (i.e., Howship’s lacunae and cement lines are absent, e.g., Figs. 3C, 3E, 3F, 4C, 4E, 5C, 7B, 7D, 9A, 10D). They likely indicate a spurt of rapid growth following a period of slow growth during the unfavorable growing season.

The observation that most of the cortex in the *Lystrosaurus* bones is comprised of primary, rapidly forming fibrolamellar bone, which is highly vascularized indicates rapid growth rates and does not reflect an osteoporotic lifestyle response comprised of secondary bone as proposed by Ray, Chinsamy & Bandyopadhyay (2005). Recent research using isotopes (Rey et al., 2017), primary osteon density (or primary osteon area) and phylogenetic eigenvector maps (Olivier et al., 2017; Faure-Brac & Cubo, 2020), and growth marks in dentine (Whitney & Sidor, 2020) have proposed that *Lystrosaurus* was endothermic. Rey et al. (2017) and Faure-Brac & Cubo (2020) suggest that all Neotherapsida (the clade that includes anomodonts and theriodonts) had an endothermic metabolism. However, it is important to state what kind of endothermy may have been present as there is a whole spectrum of metabolic states from poikilothermic ectothermy in extant reptiles to homeothermic endothermy in extant mammals and birds. It would be interesting to know where dinocephalian therapsids fit into this hypothesis as they are characterized by fibrolamellar bone with relatively large vascular canals and high vascular density (Botha & Huttenlocker, in press), but they have yet to be sampled.

Faure-Brac & Cubo (2020) refer to neotherapsid thermometabolism as being “primitively endothermic” and Whitney & Sidor (2020) suggest that *Lystrosaurus* was a heterothermic endotherm. It is highly unlikely that *Lystrosaurus* was a homeothermic endotherm as in extant mammals as there are several morphological (and physiological) features that had to be in place prior to the acquisition of homeothermic endothermy. Just two of these features include maxillary turbinates for the conservation of heat and water, and a muscular diaphragm allowing for high ventilation rates, neither of which fossilize. Ossified maxilloturbinate bones (the ridges on which the maxillary turbinate membranes rest) have been found in the Late Triassic-Early Jurassic tritheledontid *Elliotherium* and the mammaliaform *Morganucodon* (Crompton et al., 2017). Despite Laaß et al. (2011) interpreting structures in the nasal cavity of *Lystrosaurus* as cartilaginous maxilloturbinates (based on neutron tomography), this observation has never been confirmed nor has the feature been found in any other anomodont. If the maxilloturbinates remained cartilaginous during the early part of their evolution they may have been mostly involved in evaporative cooling rather than in water conservation (Crompton et al., 2017) and thus, would not have been able to support homeothermic endothermy at this stage. The presence of ribs reaching almost to the pelvic girdle and delicate nature of these bones in *Lystrosaurus* also make it unlikely that a muscular diaphragm was present (Strong, pers. comm., 2012). It is unknown when a mammal-like diaphragm evolved, but the presence of reduced lumbar ribs in the Early Triassic non-mammaliaform cynodont *Thrinaxodon* suggests that there was at least space for such a feature in this clade. Thus, it is likely that *Lystrosaurus* was at most a heterothermic endotherm as suggested by Whitney & Sidor (2020).

The irregular occurrence of growth marks in *L. murrayi* and *L. declivis* is noteworthy and suggests that these species were capable of developmental plasticity. Developmental plasticity is the ability of an organism to change its growth patterns in response to changing environmental conditions. Osteohistological developmental plasticity has been reported in extant amphibians (Alcobendas & Castanet, 2000) and birds (Starck & Chinsamy, 2002). In extinct vertebrates this feature, in the form of variation in single or double LAGs or number of LAGs and size, has been found in the Permian lissamphibian *Doleserpeton* (Gee, Haridy & Reisz, 2020) and the Triassic sauropodomorph dinosaur *Plateosaurus* (Sander & Klein, 2005). In this case, *L. murrayi* and *L. declivis* show a variable instance of LAGs at similar body sizes, similar to *Plateosaurus*. Sander & Klein (2005) found that growth mark counts were poorly correlated with body size in *Plateosaurus.* They proposed that developmental plasticity in this dinosaur indicated that it was ectothermic as strong developmental plasticity is correlated with low metabolic rates (Sander & Klein, 2005). However, there is a variety of research to suggest that dinosaurs were at least partially endothermic (e.g., Erickson et al., 2009; Grady et al., 2014; Legendre et al., 2016; Rezende et al., 2020). It is possible that *Lystrosaurus* could have varied its growth as a heterothermic endotherm. This may have been advantageous in the post-extinction Early Triassic environment, which is considered to have undergone extreme temperatures (Rey et al., 2016), and would have allowed it to undergo torpor at high latitudes as suggested by Whitney & Sidor (2020).

An overall transition to slower forming bone tissues in the form of parallel-fibered bone is only observed in the very largest *L. maccaigi* specimens. Patches of parallel-fibered bone can be found in some ontogenetically older individuals of the other species, but this bone tissue type does not dominate the cortex. Outer circumferential lamellae were not found in any specimen in this study, indicating that not even the largest *L. maccaigi* individuals had reached maximum body size. The presence of slower forming tissues in the outer periphery of the largest *L. maccaigi* bones, however, does indicate a decrease in growth rate and that these individuals were at least approaching a growth asymptote.

**Figure 11:**
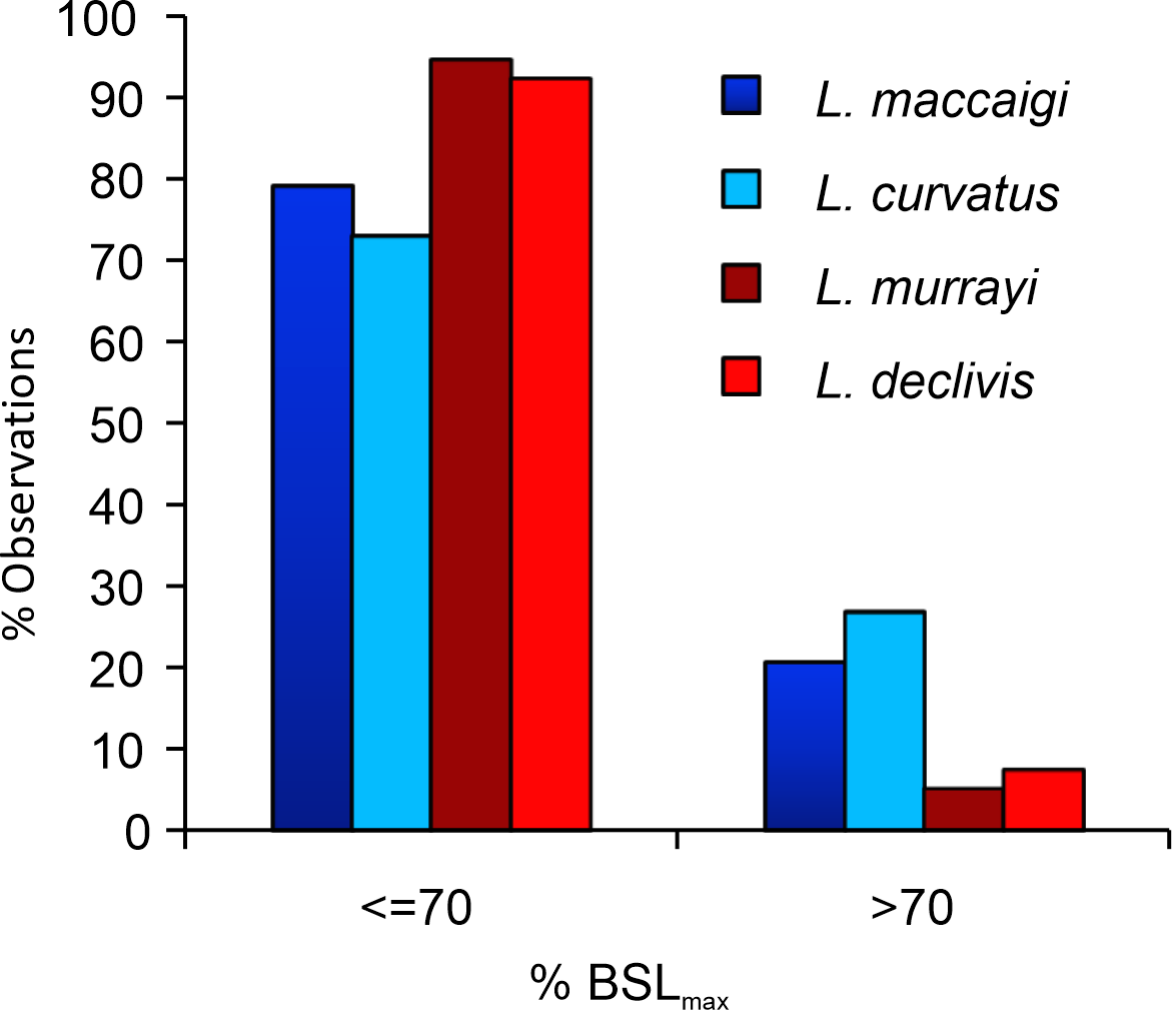
Percentage of maximum basal skull length (%BSLmax) of the four species of South African *Lystrosaurus.*. There are distinct differences in body size distributions between Permian (blue) and Triassic (red) species (taken from Botha-Brink et al., 2016).

It has long been noted that *L. murrayi* and *L. declivis* are the smaller of the four South African species (e.g., Grine et al., 2006; Botha & Smith, 2007; Botha-Brink et al., 2016) (Fig. 11; Table S2). As they are purely Triassic species it raises the possibility that *Lystrosaurus* underwent a decrease in body size across the Permo-Triassic boundary similar to the therocephalian *Moschorhinus kitchingi* (Huttenlocker & Botha-Brink, 2013). However, it is clear from the results in this study that not even the largest individuals of *L. murrayi* and *L. declivis* recovered to date had begun to reach a growth asymptote as outer circumferential lamellae or an overall transition to slower forming bone tissues are absent from all the study limb bones of these species. The basal skull length (BSL) of the largest *L. murrayi* humerus in this study (BP/1/3236), which is the largest *L. murrayi* specimen known overall, is 213 mm (Botha-Brink et al., 2016). It is 55% the size of the largest known *L. maccaigi* specimen (BP/1/4038, BSL 389 mm) and 67% the size of the *L. maccaigi* specimen in this study (NMQR 3663, BSL 319 mm) (Table S2), which exhibits a transition to slower forming bone tissues and thus a decrease in growth rate. Given the absence of such a transition in the *L. murrayi* and *L. declivis* individuals it is likely that all specimens of these species recovered to date represent juvenile or early sub-adult ontogenetic stages. Thus, these two Triassic species were capable of growing to significantly larger sizes and may even have reached similar sizes to *L. maccaigi* and *L. curvatus*. There is thus, no evidence of a Lilliput effect in *Lystrosaurus* during the EPME. The smaller BSL in *L. murrayi* and *L. declivis* are more likely the effect of shorter lifespans where these species were dying at younger ages compared to the Permian species as suggested by Botha-Brink et al. (2016). Thus, although there is evidence of a Lilliput effect in dicynodonts across the PTB with the disappearance of larger taxa (Angielczyk & Walsh, 2008) there is no evidence of a dwarfing of *Lystrosaurus* species.

The high abundance of smaller specimens in museum collections is unlikely due to a collection bias as larger specimens are easier to find than smaller specimens. There is a change in taphonomic style from the Permian to Triassic where more articulated skeletons are found in Triassic deposits, but skull material is found equally in both deposits (Smith & Botha-Brink, 2014). The burrowing lifestyle of *Lystrosaurus* may also have increased its preservational potential, however, even Permian species of *Lystrosaurus* (e.g., *L. curvatus*, Botha-Brink, 2017) were capable of burrowing. Furthermore, a burrowing lifestyle should not bias the preservation of smaller specimens as *Lystrosaurus* is considered to have burrowed throughout its entire lifespan. Thus, even large specimens should preserve as easily as smaller individuals. The abundance of smaller individuals of *Lystrosaurus* in the *Lystrosaurus declivis* Assemblage Zone is likely a real biological phenomenon and not one of taphonomic bias.

A semi-aquatic lifestyle for *Lystrosaurus* was first proposed by Broom (1902) and Broom (1903) and later supported by several studies (Watson, 1912; Watson, 1913; Brink, 1951; Camp, 1956; Cluver, 1971; Hotton, 1986; Kemp, 1982; Kitching, 1968) based on various cranial and postcranial morphological features. These studies were later refuted by King (1991) and King & Cluver (1991) who proposed that the morphological characteristics used to infer an aquatic lifestyle could equally represent a fossorial or burrowing ecology. Later studies (Smith & Botha, 2005; Botha & Smith, 2007) suggested that an aquatic lifestyle was unlikely given the high abundance and success of *Lystrosaurus* in an increasingly arid environment. However, Ray, Chinsamy & Bandyopadhyay (2005) resurrected the aquatic hypothesis based on the microanatomy of *Lystrosaurus* limb bones and ribs where they suggested that the trabecular infilling of the limb bone medullary cavities and high cortical thickness in the ribs was indicative of an amphibious lifestyle.

The most recent study to predict the lifestyle of *Lystrosaurus* is Canoville & Laurin (2010) who used a humerus in Bone Profiler and found it to predict an amphibious lifestyle. However, Kriloff et al. (2008) also proposed an amphibious lifestyle for *Dicynodon* using the Bone Profiler program, a taxon that is considered to be fully terrestrial based on its morphology. When Bone Profiler was used in this study to predict the lifestyle of *Lystrosaurus* the program also predicted an aquatic lifestyle for all four species. This appears to be based on the trabecular infilling of the medullary cavities. Fully aquatic, pelagic taxa generally exhibit medullary cavities filled with bony trabeculae and broad transition zones that result in a mostly cancellous or very narrow cortex (e.g., the dolphin, *Delphinus delphis*), whereas amphibious shallow water taxa tend to have very thick compact cortices, which help to counteract buoyancy while in the water (e.g., the manatee, *Dugong dugong*) (Canoville & Laurin, 2010). The infilled medullary cavities in the limb bones of *Lystrosaurus* would suggest a fully aquatic pelagic lifestyle, but the morphology of this genus (and dicynodonts as a clade) is inconsistent with a pelagic ecology and rather suggests a fully terrestrial mode of life. Given that the genus *Lystrosaurus* was a relatively large taxon (and that all four species may have grown equally large), it is possible that the trabecular infilling increased the overall strength of the bones and would have been a biomechanical advantage in a fossorial animal. Trabecular infilling of limb bone medullary cavities has been observed in several other dicynodont taxa such as *Rhachiocephalus*, *Aulacephalodon*, *Daptocephalus* and *Kannemeyeria* (Botha-Brink & Angielczyk, 2010; Ray, Botha-Brink & Chinsamy, 2012) and may have thickened the bones and acted as buttresses against the weight of these animals.

Given these results, it is thus worth cautioning against using Bone Profiler as the only tool to infer lifestyle for anomodonts. Other studies have found similar issues with using bone microanatomy to infer lifestyle for the pelycosaur *Ophiacodon* (see discussions in Germain & Laurin, 2005; Felice & Angielczyk, 2014; Laurin & De Buffrenil, 2016; Huttenlocker & Shelton, 2020). The biomechanics of therapsids have no modern analogues with respect to stance and locomotory gait (e.g., the sprawling forelimbs and erect hind limbs), and thus, other factors may have played a role in shaping the bone microanatomy in anomodont therapsids.

### Conclusion

The highly vascularized fibrolamellar bone present in all four species of South African *Lystrosaurus* indicates that the genus grew rapidly during early to mid-ontogeny, similar to many other Permo-Triassic dicynodonts. There is no notable difference in bone tissues between the four species, except that growth marks are rare and inconsistently present in *L. murrayi* and *L. declivis* specimens of similar body size. This suggests that developmental plasticity may have been present in these taxa and may have been advantageous in the unpredictable post-extinction Early Triassic environment. A transition to slower forming parallel-fibered bone can be seen in older individuals of Permian *L. maccaigi*. The absence of slower forming bone tissues or outer circumferential lamellae in *L. curvatus*, *L. murrayi* and *L. declivis* indicates that even the largest specimens were not close to a growth asymptote. Thus, there is no evidence of a Lilliput effect in South African *Lystrosaurus* in the form of dwarfing of the Triassic species, contrary to another EPME survivor, the therocephalian *Moschorhinus kitchingi*.

Despite some previous studies suggesting that *Lystrosaurus* was aquatic, this study supports a terrestrial mode of life. The trabecular infilling of the medullary cavities is similar to that found in some other large dicynodonts with no morphological aquatic characteristics. Medullary cavity infilling may have been a biomechanical mechanism for supporting the weight of these animals.

##  Supplemental Information

10.7717/peerj.10408/supp-1 Supplemental Information 1 Osteohistological parameters of *L. maccaigi*, *L. curvatus*, *L. murrayi* and *L. declivis* Cranial measurements, primary osteon diameter, vascularization, dominant bone tissue type and growth mark count of the four South African *Lystrosaurus* species. Abbreviations: pod, primary osteon diameter; vasc, vascularization.

## Additional Information and Declarations

### Data Availability

The following information was supplied regarding data availability: The raw measurements are available in the Supplemental File.
